# PERINEAL REPAIR AFTER ABDOMINOPERINEAL EXCISION WITH RECTUS ABDOMINIS MYOCUTANEOUS FLAP

**DOI:** 10.1590/0102-672020190001e1507

**Published:** 2020-11-20

**Authors:** Elsa D’ANNUNZIO, Alain VALVERDE, Renato Micelli LUPINACCI

**Affiliations:** 1 Department of Digestive Surgery, Diaconesses Croix Saint Simon Hospital, Paris, France

**Keywords:** Abdominoperineal resection, Perineal wound, Rectus abdominis flap, Excisão abdominoperineal do reto, Períneo, cirurgia, Retalho muscular

## Abstract

**Background:**

Abdominoperineal excision of the rectum (APR) remains the only potential curative treatment for very low rectal adenocarcinoma and squamous cell carcinoma of the anus. Yet, it implies a significant perineal exenteration and has set the attention on the perineal reconstruction.

**Aim:**

To present technique used in one case of APR for anal cancer, with resection of the vaginal posterior wall with large perineal defect which has called for the necessity of a flap for reconstruction

**Method:**

To cover the large perineal defect and reconstruct the posterior vaginal wall was perform a standardized and reproducible surgical technique using oblique rectus abdominis myocutaneous (ORAM) flap. The overlying skin of this flap is thick and well vascularized by both superficial branches and perforators of the superior epigastric artery and the deep inferior epigastric artery which serves as the vascular pedicle for the ORAM flap.

**Results:**

This procedure was applied in a 65-year-old woman with recurrent squamous cell carcinoma of the anus infiltrating the posterior wall of the vagina. Was performed an APR with en-bloc resection of the vaginal posterior wall in order to achieve tumor-free margins. Postoperative course was uneventful and she was discharged home at postoperative day 9. Final pathological report confirmed the oncological adequacy of the procedure (R0) and showed a rypT4N0 lesion.

**Conclusion:**

Flap reconstruction is an effective way to cover the perineal wound reducing both perineal complication rate and wound healing delay. The ORAM is particularly interesting for female whose tumors require resection and subsequent reconstruction of the posterior wall of the vagina.

## Graphical Abstract

[Fig f1a]

**Figure f1a:**
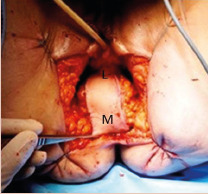
Myocutaneous flap of the rectus abdominis filling the perineal defect after APR.

## RESUMO

**Racional:**

A amputação abdominoperineal do reto (APR) continua sendo o único tratamento curativo nos casos de adenocarcinoma retal muito baixo e carcinoma espinocelular do ânus. No entanto, implica em exenteração perineal significativa e exige atenção na reconstrução perineal.

**Objetivo:**

Propor, ilustrando com um caso clinico, proposta de APR para câncer anal com ressecção da parede posterior da vagina com cobertura do grande defeito perineal através de reconstrução com retalho miocutâneo oblíquo do reto abdominal (ORAM).

**Método:**

Para cobrir o defeito e reconstruir a parede vaginal posterior, realizou-se técnica de retalho miocutâneo oblíquo do reto abdominal (ORAM). A pele subjacente desse retalho por ser espessa e bem vascularizada por ramos perforantes superficiais da artéria epigástrica superior e pela artéria epigástrica inferior profunda serviu como pedículo vascular.

**Resultado:**

Este procedimento foi aplicado em uma mulher de 65 anos com recidiva de carcinoma espinocelular do ânus infiltrado na parede posterior da vagina. Foi realizada APR com ressecção em bloco da parede posterior vaginal com o objetivo de obter margens livres de tumor. O pós-operatório transcorreu sem intercorrências e a paciente recebeu alta hospitalar no 9° dia pós-operatório. O relatório patológico final confirmou a adequação oncológica do procedimento (R0) e mostrou uma lesão rypT4N0 de 6,5 cm.

**Conclusão:**

A reconstrução perineal com utilização de retalho é maneira eficaz de fechar a ferida operatória, reduzindo a taxa de complicações perineais e o atraso na cicatrização. O retalho de tipo ORAM é particularmente interessante para mulheres cujos tumores requerem ressecção e subsequente reconstrução da parede posterior da vagina.

## Graphical Abstract

[Fig f1as]

**Figure f1as:** Retalho miocutâneo do reto abdominal preenchendo o defeito perineal apos APR

## INTRODUCTION

Abdominoperineal excision of the rectum (APR) remains the only potential curative treatment for very low rectal adenocarcinoma and squamous cell carcinoma of the anus^10^. It implies a significant perineal exenteration leaving a large defect usually surrounded by scar tissue damaged by radiotherapy. The perineal wound management is challenging and several options remain to deal with it, ranging from direct closure to flap reconstruction*.* The former is associated with specific complications such as pain, delayed or non-healing wound, hemorrhage and infection, perineal fistula or sinus, and secondary perineal hernia^3^. This high morbidity results in prolonged postoperative hospital stay, hospital readmissions, and homecare nursing representing a heavy healthcare cost^11^. 

Plastic surgical reconstruction of the perineum is an effective way to cover wound, thus reducing both perineal complication rate and wound healing delay^6^. Several options for reconstruction are already commonly performed by plastic surgeons, including vertical or oblique rectus abdominis myocutaneous flap (ORAM), gluteal fasciocutaneous flap and gracilis myocutaneous flap^5^ ^,^ ^8^ ^,^ ^9^using rectus abdominus (RAM. A flap is particularly interesting for female whose tumors require resection and subsequent reconstruction of the posterior wall of the vagina^1^. 

In our surgical department, digestive surgeons have chosen to perform themselves the ORAM reconstruction after APR in order to simplifying patient management and allowing operating room scheduling optimization. 

The objective of this work was to detail our standardized and reproducible surgical technique of ORAM. 

## METHOD

The manuscript was submitted and approved by the GHDCSS Research Ethics Committee and the patient has signed an informed consent form.

### Surgical technique

#### Anatomy

The rectus abdominis muscles are encased in a sheath composed of the aponeuroses of the lateral abdominal muscles. The overlying skin is thick and well vascularized by both superficial branches and perforators of the superior epigastric artery and the deep inferior epigastric artery (DIEA), a branch of the external iliac artery, and serves as the vascular pedicle for the ORAM flap. This vessel is almost universally present and is rarely affected by atherosclerotic disease^1^. However**,** we routinely ask our radiologist to confirm, on a CT scan arterial phase, that the artery is present and permeable. 

#### Harvesting of the flap at the donor site

**FIGURE 1:**
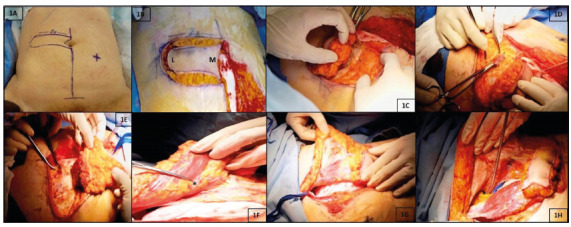
A and B) Incision follows the marked line, including fat and subcutaneous fat, flush with the anterior sheath of the external oblique muscle and skin undermining is carried up to the rectus sheath; C) incision of the anterior layer of the rectus sheath is performed sequentially: proximally, distally, laterally (L) to the flap (observing 1 cm margin larger than the skin patch), and medially (M) (close to the midline); D) rectus abdominis muscle is then cut proximally to the flap, at this point hemostasis of superior epigastric vessels must be secured; E and F) once the muscle is cut, posterior aspect of the muscle can be freed from its sheath; stitches between fascia and skin are temporary put to avoid slippage of the skin cover during dissection which carried on further down and intercostal pedicules encountered are to be ligated (*indicates the DIEA pedicle); G) anterior aspect of the rectus muscle is then totally freed from its sheath; H) the flap is totally mobilized (*indicates the DIEA pedicle)

The flap is designed and marked on the patient, axis following the line between umbilicus and tip of the right scapula (Figure 1A). The flap should be large enough to fill in the wound defect and associated dead space, but it should also result in minimal donor site morbidity. Width of the flap must permit skin closure. Therefore, it must be respected a ratio length/width of 2:1. The maximal theoretical size of the flap is 30:20 cm but we already performed it with 16:8 cm, 18:9 cm or 20:10 cm flap, which is usually large enough for recovering the perineal defect. 

Incision follows the marked line*,* including skin and subcutaneous fat and is carried up to the rectus sheath (Figure 1B)*.* The anterior layer of the rectus sheath is cut sequentially: proximally, distally and laterally to the flap (Figure 1C). It is important to observe a 1 cm margin larger than the skin patch at the upper, lower and lateral sides. The rectus abdominis muscle is then cut proximally to the flap (Figure 1D). At this point, hemostasis of superior epigastric vessel must be secured and some stitches between the anterior fascia and the flap skin are temporary put to avoid slippage of the skin cover which may compromise the flap viability.

After the muscle has been cut at the upper part of the flap, we can easily free the posterior aspect of the muscle from its sheath (Figures1E and 1F)*.* This dissection is carried on further down. Intercostal pedicles encountered during that dissection part are to be ligated. Anterior aspect of the bottom part of rectus muscle is then totally dissected from its anterior sheath (Figure 1G). Finally, the posterior aspect of the muscle is then dissected further, progressing above the arcuate line (linea semicircularis) until groin (Figure 1H). Dissection of the DIEA up to the external iliac vessel can be performed in need of more length. In that case, muscle is inferiorly detached from the pubis. This complementary dissection is occasionally recommended by plastic surgeons, but in our experience it is not necessary and may put at risk the flap vascularization. 

#### Perineal reconstruction

**FIGURE 2:**
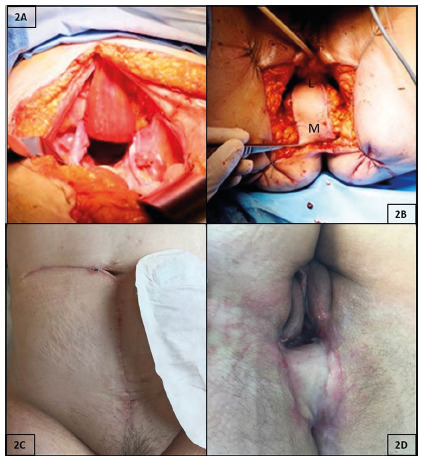
A) The flap is rotated 180° clockwise on its DIEA pedicle and is tunneled via intraperitoneal route into the pelvis (care must be taken not to kink or place tension on the vascular pedicule); B) the flap is led precautiously to the perineal defect in order that the lateral end of the flap (L) covers the anterior side of the defect and temporary stitches that were placed between aponeurosis and skin are cut, flap is positioned and then sewed cutaneocutanously with separated resorbable stitches (in case of posterior vaginal resection, the flap will satisfactorily replace it); C and D) aspect of the abdominal wall and the perineum six weeks after surgery.

The harvested rectus flap is rotated 180° in a clockwise manner on its DIEA pedicle to reach the pelvis (Figure 2A). Care must be taken not to kink or place tension on the vascular pedicle. The flap is rotated and led precautiously to the perineal defect in order that the lateral end of the flap covers the anterior side of the defect (Figure 2B). Closure of the perineal defect is performed by interrupted vertical mattress suture using resorbable polyglactine stitches. In case of posterior vaginal defect, is recommended to start the perineal closure by putting some stitches between the skin flap and the remaining walls of the vagina in order to create an adequate neovagina. 

#### Closure of the abdominal wall at the donor site

Median line can be steadily and tension free sutured with slow resorbable stitches since all its layers are intact. In case of necessity, it can be used polyglactin 910 mesh. 

### Specific postoperative care

Abdominal wall closure and flap viability are to be checked every day and covered by vaseline gauze. Patient should not sit nor walk for five to seven days and thus physiotherapy is of uttermost importance. Urinary catheter is kept at least for five days. 

## RESULTS

This procedure was applied in a case of 65-year-old woman with recurrent squamous cell carcinoma of the anus infiltrating the posterior wall of the vagina. After multidisciplinary staff decision, we performed an abdominoperineal excision of the rectum with en-bloc resection of the vaginal posterior wall in order to achieve tumor-free margins. It resulted in a large perineal defect surrounded by scar tissue damaged by radiotherapy. In the present case, the whole posterior wall of the vagina could be reconstructed.

She was asked to remain on bed rest for five days following surgery and was encouraged to be out of bed and ambulating on the sixth day. Postoperative monitoring was done exclusively by clinical examination (color and time for recapilarization). Postoperative course was uneventful and she was discharged home from hospital at postoperative day 9. Final pathologic report showed a rpT4N0 6.5x6.5cm squamous cell carcinoma with invasion of the vaginal submucosa. Resection was considered R0. 

At six-weeks after surgery, clinical examination showed a solid anterior abdominal wall (Figure 2C) and a very satisfying vaginal reconstruction allowing the potential for normal function, i.e.: 1) vagina was >10 cm long; 2) vaginal opening easily admitted two fingers during examination; 3) the relationship of the posterior vagina and the perineum was almost perpendicular, and 4) there was no buildup of perineal skin above and beyond the flap. 

## DISCUSSION

APR remains the appropriate approach for many situations, such as cancers that involve the sphincter complex or that cannot be removed with an adequate distal resection margins, and for elderly adults with poor baseline functional status^4^”page”:”1477-1487”,”volume”:”22”,”issue”:”8”,”source”:”PubMed”,”abstract”:”BACKGROUND: Management of low rectal cancer continues to be a challenge, and decision making regarding the need for an abdominoperineal resection (APR. Also, surgery represents the recommended therapy for persistent or recurrent anal canal cancer after chemoradiotherapy treatment^7^. The increasing utilization of pelvic radiotherapy in the treatment of anal and rectal carcinomas and the adoption of the cylindrical extralevator abdominoperineal excision lead to larger perineal defects and pelvic dead space which can result in considerable morbidity from these perineal wounds^7^. Reported incidences of perineal wound problems vary widely, but have been observed in up to 47% of patients following APR, leading to intensive wound care, prolonged hospital stay, and a diminished quality of life^1^ ^,^ ^2^. Several strategies have been developed to deal with the problem. The use of autologous tissue transfer is interesting to obliterate the pelvic dead space, thereby preventing presacral abscess formation, and the positive influence a well-vascularized tissue might have on wound healing, especially after radiotherapy^2^. 

Despite several techniques currently employed for perineal closure after APR, it still remains unclear as to which strategy is superior. Omentoplasty was the first choice for colorectal surgeons for many years because of its simplicity, but has unsatisfactory results and may be an independent risk factor for perineal hernia formation^2^. Rectus abdominis muscle flaps have the advantage of utilizing the primary incision, require only one donor site, and have a vascular pedicle with a large arc of rotation that is highly reliable. A recent meta-analysis found the overall rate of any perineal wound or flap complication among rectus abdominis muscle patients to be significantly lower than gluteal and gracilis flap^5^. Although commonly performed by plastic surgeons, we have decided to perform perineal flap reconstruction by ourselves and, in our experience, it has simplified patient’s management and operating room scheduling. 

## CONCLUSION

This technique of perineal repair with rectus abdominis myocutaneous flap after abdominoperineal excision is safe, reproducible and offers good long-term results.
